# Highly efficient dual-wavelength mid-infrared CW Laser in diode end-pumped Er:SrF_2_ single crystals

**DOI:** 10.1038/srep36635

**Published:** 2016-11-04

**Authors:** Weiwei Ma, Xiaobo Qian, Jingya Wang, Jingjing Liu, Xiuwei Fan, Jie Liu, Liangbi Su, Jun Xu

**Affiliations:** 1 Synthetic Single Crystal Research Center, Shanghai Institute of Ceramics, Chinese Academy of Sciences, No.588 Heshuo Road, Shanghai 201899, China; 2 Key Laboratory of Transparent and Opto-functional Inorganic Materials, Shanghai Institute of Ceramics, Chinese Academy of Sciences, Shanghai 201800, China; 3 University of Chinese Academy of Sciences, No.19A Yuquan Road, Beijing 100049, China; 4 Institute of Data Science and Technology, School of Physics and Electronics, Shandong Normal University, Jinan 250014, China; 5 School of Physics Science and Engineering, Institute for Advanced Study, Tongji University, Shanghai 200092, China

## Abstract

The spectral properties and laser performance of Er:SrF_2_ single crystals were investigated and compared with Er:CaF_2_. Er:SrF_2_ crystals have larger absorption cross-sections at the pumping wavelength, larger mid-infrared stimulated emission cross-sections and much longer fluorescence lifetimes of the upper laser level (Er^3+^:^4^I_11/2_ level) than those of Er:CaF_2_ crystals. Dual-wavelength continuous-wave (CW) lasers around 2.8 μm were demonstrated in both 4at.% and 10at.% Er:SrF_2_ single crystals under 972 nm laser diode (LD) end pumping. The laser wavelengths are 2789.3 nm and 2791.8 nm in the former, and 2786.4 nm and 2790.7 nm in the latter, respectively. The best laser performance has been demonstrated in lightly doped 4at.% Er:SrF_2_ with a low threshold of 0.100 W, a high slope efficiency of 22.0%, an maximum output power of 0.483 W.

Er^3+^ ion based laser materials operating at around 2.8 μm have attracted increasing interest in recent years because of their various important applications^1^,^2^,^3^,^4^. Lasers around this specific region can be utilized for medical surgery, dentistry, remote sensing etc, because of the strong water absorption around this spectral region^5^,^6^. Moreover, lasers around 2.8 μm are suitable pumping source for far-infrared optical parametric oscillation (OPO), which have broad applications in scientific research, atmospheric pollution monitoring, and directional infrared countermeasure^7^.

However, the intrinsic self-terminating effect of Er^3+^ ion impedes the improvement Er^3+^ ion based mid-infrared lasers. Because the fluorescence lifetime of the initial laser level (Er^3+^:^4^I_11/2_) is considerably shorter than that of the terminal laser level (Er^3+^:^4^I_13/2_), and thus will lead to high threshold, low slope efficiency and even self-terminating of continuous-wave (CW) laser. One major solution to suppress this detrimental effect is to increase Er^3+^ doping concentration. One key point of this solution is that with the increase of Er^3+^ concentration, the lifetime of the Er^3+^:^4^I_13/2_ level quenches faster than that of the Er^3+^:^4^I_11/2_ level, thus narrow the lifetime gap of these two levels^3^,^8^. Another key point of high Er^3+^ doping is to enhance the energy transfer process between Er^3+^ ions by shortening the Er^3+^ spacing with high doping concentration. However, the drawback of high Er^3+^ doping is that the crystal quality and thermal conductivity will degenerate significantly with the increasing of Er^3+^ concentration. Co-doping deactivating ions, such as Pr^3+ ^1,6,7,9, Nd^3+ ^2,10, Ho^3+ ^11 etc, is another way to overcome this “bottleneck” effect by effectively depopulating the Er^3+^:^4^I_13/2_ level, but only a few have achieved laser output. Furthermore, after co-doping deactivating ions the theoretical slope efficiency limit will be lower, because part of the energy of Er^3+^ ions is depleted by deactivating ions^12^,^13^. And deactivating ions also will have inevitable negative effect on Er^3+^:^4^I_11/2_ level due to complicated energy transfer process^1^,^9^. Hence, host materials that can achieve mid-infrared CW laser under low Er^3+^ doping concentration would be more favorable.

In Er^3+^ based oxide crystals, such as Y_3_Al_5_O_12_ (YAG)^11^,^14^,^15^, Gd_3_Ga_5_O_12_ (GGG)^16^ and Gd_3−x_Y_x_Sc_2_Ga_3_O_12_ (GYSGG)^1^,^7^ etc, the Er^3+^ doping concentrations vary from 30 at.% to as high as 50 at.%. Lu_2_O_3_ crystal has the lowest Er^3+^ doping concentration of 7at.% among that group^8^. Such high doping concentration not only significantly decreases the thermal conductivity of crystal but also leads to extremely short lifetime of the upper laser level (Er:^4^I_11/2_)^8^,^14^. Compared with oxide crystals mentioned above, SrF_2_ crystal same as CaF_2_ crystal have an key advantage that is rare earth ions tend to form clusters even when the doping concentration is low^17^,^18^. Such clusters additional shorten the distance between Er^3+^ ions and thus enhances the energy transfer process between them, which is beneficial for achieving mid-infrared laser under low doping concentration. In addition, SrF_2_ crystal has an even lower phonon energy of ~280 cm^−1^ than ~322 cm^−1^ of CaF_2_ crystal^19^,^20^, which is much lower than that of most oxide crystals like YAG (700 cm^−1^)^21^. Low phonon energy plays an important role in reducing the probability of non-radiative transition, prolonging the fluorescence lifetime of the upper laser level and improving the laser performance.

Until now the highest reported slope efficiency (η) from Er:CaF_2_ crystal is 30%^5^ pumped by a Ti:sapphire laser, which is comparable with those best results reported in other famous Er-doped crystals. Such as 30at,% Er:Gd_3_Sc_2_Ga_3_O_12_ (GSGG) (η = 36%)^22^, 15at.% Er:LiYF_4_ (η = 35%)^23^, 50at.% Er:YAG (η = 34%)^24^, and 7at.% Er:Lu_2_O_3_ (η = 36%)^8^. However, the highest reported slope efficiency from diode-pumped Er:CaF_2_ crystal is much lower (η = 4%)^25^. There is less reports about Er:SrF_2_ crystal and the highest reported slope efficiency is 11%^25^,^26^.

This work we aim to investigate and compare the spectral properties and laser performance of Er:SrF_2_ and Er:CaF_2_ single crystals. We also report dual-wavelength continuous-wave lasers around 2.8 μm in both 4at.% and 10at.% Er:SrF_2_ single crystals under 972 nm laser diode (LD) end pumping. 4at.% Er:SrF_2_ demonstrated the best laser performance with a low threshold of 0.100 W, a high slope efficiency of 22.0% and an maximum output power of 0.483 W.

## Materials and Methods

4at.% Er:SrF_2_, 10at.% Er:SrF_2_, 4at.% Er:CaF_2_ and 11at.% Er:CaF_2_ crystals were grown by traditional vertical Bridgman method, using high purity (>99.995%) ErF_3_, SrF_2_ and CaF_2_ crystalline powders as raw materials. The raw materials were ground, mixed, and sealed with additional deoxidant in platinum crucibles during the whole process of growth. The crystal samples were cut and then polished into a size of 10 × 10 × 1.0 mm^3^ for spectral measurements.

The actual concentrations of Er^3+^ ions in the grown crystals were measured by inductively coupled plasma atomic emission spectroscopy (ICP-AES) method. Absorption spectra were measured by a Cary 5000 UV/VIS/NIR spectrophotometer. 1.5 μm, 2.7 μm emission as well as the fluorescence lifetimes of Er^3+^:^4^I_13/2_ level were measured with an Edinburgh FLSP920 fluorescence spectrophotometer, using an 980 LD as excitation source. And the fluorescence lifetimes of Er^3+^:^4^I_11/2_ level were measured with an Edinburgh FLS920 fluorescence spectrophotometer, using an ns OPO laser, emitting at 980 nm, as excitation source. All the measurements were carried out at room temperature.

## Results and Discussion

### Ions concentrations

The actual concentrations of Er^3+^ ions in these samples were measured by ICP-AES. The values were 7.96 × 10^20^, 21.84 × 10^20^ cm^−3^ for 4at.% ErSrF_2_ and 10at.% Er:SrF_2_, respectively. And 9.72 × 10^20^, 25.84 × 10^20^ cm^−3^ for 4at.% Er:CaF_2_ and 11at.% Er:CaF_2_, respectively.

### Absorption spectra

**Figure 1:**
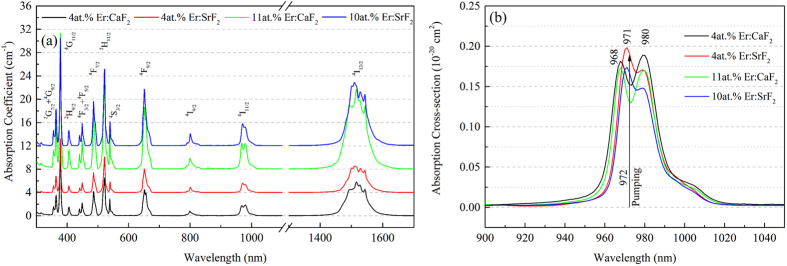
Absortption spectra (**a**) and cross-sections (**b**) of 4at.% Er:SrF_2_, 10at.% Er:SrF_2_, 4at.% Er:CaF_2_ and 11at.% Er:CaF_2_ crystals at room temperature.

The absorption spectra of four crystals, namely 4at.% Er:SrF_2_, 10at.% Er:SrF_2_, 4at.% Er:CaF_2_ and 11at.% Er:CaF_2_, were illustrated in Fig. 1a. Within the range from 300 nm to 1700 nm, 13 main absorption bands of Er^3+^ corresponding to the transitions from ground state Er^3+^:^4^I_15/2_ to higher levels were marked in Fig. 1a. The absorption spectra of Er:SrF_2_ and Er:CaF_2_ had same numbers of bands located in similar positions. Only the shape of two absorption bands, corresponding to the transition of Er^3+^:^4^I_15/2_ to Er^3+^:^4^I_11/2_ and Er^3+^:^4^I_15/2_ to Er^3+^:^4^I_13/2_, were slightly different.

With the absorption coefficients and the actual concentrations of Er^3+^, the absorption cross-sections around 980 nm were calculated and shown in Fig. 1b. From Fig. 1b, the differences of this band between Er:SrF_2_ and Er:CaF_2_ crystals could be seen clearly. Er:CaF_2_ crystals had two quite even peak points located in 968 nm and 980 nm, respectively. While Er:SrF_2_ crystals had a slightly narrower band than Er:CaF_2_ crystals and the peak values of 971 nm were significantly higher than that of 980 nm. The detailed values of the absorption coefficients (α), the absorption cross-sections (σ_abs_) and the full widths at half-maximum (FWHM) of these bands were listed in Table 1.

In this research, an InGaAs LD emitting at 972 nm was employed as the pumping source when proceeding the mid-infrared continuous-wave (CW) laser experiments. Hence, under this pumping condition Er:SrF_2_ crystals had an advantage over Er:CaF_2_ crystals.

### Emission spectra

The mid-infrared (MIR, 2500–2860 nm) emission spectra of 4at.% Er:CaF_2_, 11at.% Er:CaF_2_, 4at.% Er:SrF_2_ and 10at.% Er:SrF_2_ crystals were measured with 980 nm LD excitation under the same condition. Both Er:CaF_2_ and Er:SrF_2_ crystals had broad MIR emission bands, which were beneficial for achieving ultra-short pulse laser and tunable laser operation.

**Figure 2:**
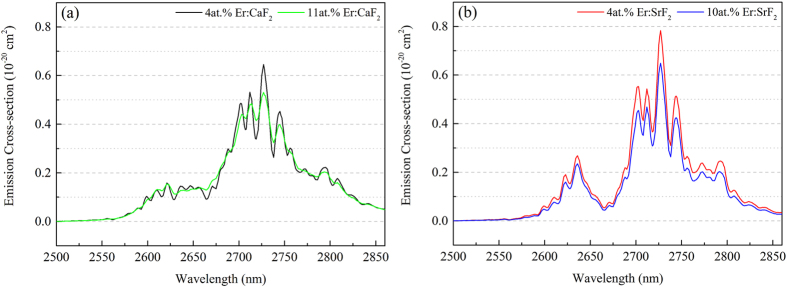
MIR stimulated emission cross-sections of 4at.% Er:CaF_2_, 11at.% Er:CaF_2_ (**a**), 4at.% Er:SrF_2_ and 10at.% Er:SrF_2_ (**b**) crystals at room temperature.

Based on the Fuchtbauer-Ladenburg equation^27^,^28^ and the MIR emission spectra, the MIR stimulated emission cross-sections were calculated and illustrated in Fig. 2:


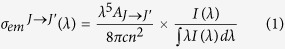


where λ is the wavelength, *I*(λ) is the intensity of emission spectrum, *I*(λ)/∫λ*I*(λ)dλ is the profile function of emission spectrum, c is the speed of light in vacuum, n is the refractive index, and *A*_*J*′*→J*′_ (*J* = ^4^I_11/2_, *J*′ = ^4^I_13/2_) is the corresponding radiative transition probability, which is calculated by Judd-Ofelt theory from the absorption spectrum^28^. If the emission spectrum, refractive index and radiative transition probability are known, then the emission cross-section can be calculated from equation 1.

Four calculated emission cross-sections (σ_em_) at 2727 nm were 0.78 × 10^−20^, 0.65 × 10^−20^, 0.65 × 10^−20^, 0.53 × 10^−20^ cm^2^ for 4at.% Er:SrF_2_, 4at.% Er:CaF_2_, 10at.% Er:SrF_2_ and 11at.% Er:CaF_2_, respectively. It was obvious that 4at.% Er:SrF_2_ had the highest value of σ_em_ (0.78 × 10^−20^ cm^2^ at 2727 nm), which was 20% higher than that of 4at.% Er:CaF_2_. High emission cross-section was favorable in achieving high performance of MIR laser operation.

**Figure 3:**
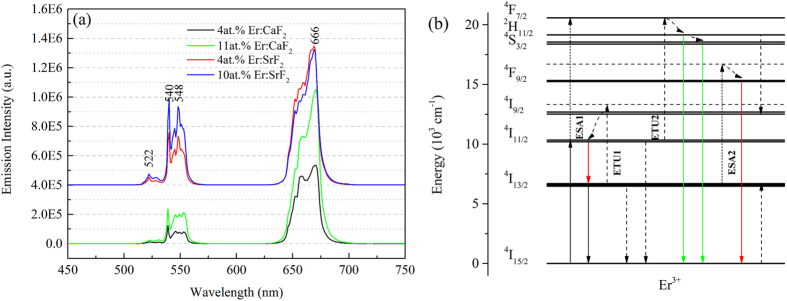
Up-conversion emission spectra at room temperature (**a**) and energy level diagram of Er^3+^ ions (**b**).

Figure 3a showed the up-conversion emission spectra of these four crystals measured under 980 nm LD excitation at room temperature. Both up-conversion emission spectra of Er:CaF_2_ and Er:SrF_2_ contained green and red emission bands, corresponding to Er^3+^:^2^H_11/2_ + ^4^S_3/2_ → ^4^I_15/2_ and Er^3+^:^4^F_9/2_ → ^4^I_15/2_. However, obvious differences could be told from Er:CaF_2_ and Er:SrF_2_. Firstly, in Er:CaF_2_ with the increase of Er^3+^ concentration both green and red up-conversion increased quite evenly. While in Er:SrF_2_ only green up-conversion increased with the increase of Er^3+^ concentration. Secondly, the proportion of green and red emission was different in Er:CaF_2_ and Er:SrF_2_. The proportion of green and red emission were approximately 1:7.8 and 1:5.7 for 4at.% Er:CaF_2_ and 11at.% Er:CaF_2_, respectively. And for 4at.% Er:SrF_2_ and 10at.% Er:SrF_2_ were 1:4.1 and 1:2.3, respectively.

In order to understand the up-conversion emission spectra of Er:SrF_2_ and Er:CaF_2_, the energy level diagram of Er^3+^ was presented in Fig. 3b. The possible mechanisms of energy transfer processes between Er^3+^ ions were illustrated, which were similar to that proposed by C. Labbe^5^. Two excited state absorption (ESA) processes, namely ESA 1 and ESA 2. Two energy transfer up-conversion (ETU) processes, namely ETU 1 and ETU 2. And one cross relaxation process (CR). ESA 1: Er^3+^:^4^I_11/2_ + hv → ^4^F_7/2_. ESA 2: Er^3+^:^4^I_13/2_ + hv → ^4^F_9/2_. ETU 1: Er^3+^:^4^I_13/2_ + ^4^I_13/2_ → Er^3+^:^4^I_15/2_ + ^4^I_11/2_. ETU 2: Er^3+^:^4^I_11/2_ + ^4^I_11/2_ → Er^3+^:^4^I_15/2_ + ^4^F_7/2_. CR: Er^3+^:^4^S_3/2_ + ^4^I_15/2_ → Er^3+^:^4^I_9/2_ + ^4^I_13/2_.

Among these energy transfer processes, ESA 1 and ETU 2 were detrimental because they reduced the population of the upper laser level (Er^3+^:^4^I_11/2_) and then resulted in the green emission. It was worth noting that the phonon energy of CaF_2_ and SrF_2_ were about 322 cm^−1^ and 280 cm^−1^. And the energy gap between Er^3+^:^4^S_3/2_ and ^4^F_9/2_ was about 3000 cm^−1^. Thus the non-radiative transition probability between Er^3+^:^4^S_3/2_ and ^4^F_9/2_ should be quite low. So ESA 2 should be the major process that was responsible for the red emission. ETU 1 was beneficial because it deactivated the lower laser level (Er^3+^:^4^I_13/2_) and partly repopulated the upper one (Er^3+^:^4^I_11/2_), which was the key to suppress the self-termination problem in Er^3+^ singly-doped crystals. As could be seen that both ETU 1 and ESA 2 relied on the population of lower laser level (Er^3+^:^4^I_13/2_), so strong red emission would have negative effect on ETU 1 process. In conclusion, both green and red up-conversion emission were detrimental to achieving MIR laser operation.

Hence, samples with weaker green and red up-conversion emission intensity would be more suitable for MIR laser operation. 4at.% Er:SrF_2_ and 4at.% Er:CaF_2_ crystals might have better laser performance than the other two highly doped crystals.

### Fluorescence lifetime

**Figure 4:**
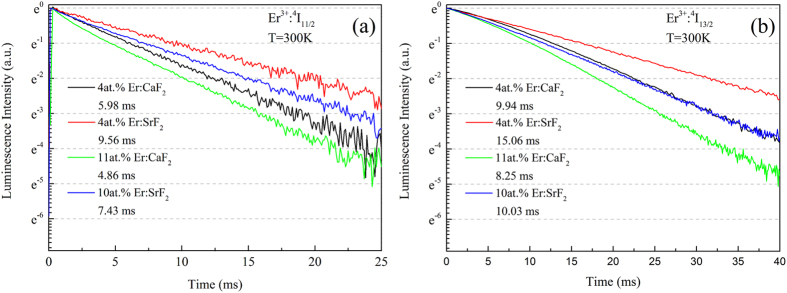
Decay curves of Er^3+^:^4^I_11/2_ level (**a**) and Er^3+^:^4^I_13/2_ level (**b**) in 4at.% Er:SrF_2_, 10at.% Er:SrF_2_, 4at.% Er:CaF_2_ and 11at.% Er:CaF_2_ crystals at room temperature.

The fluorescence decay curves of Er^3+^:^4^I_11/2_ and Er^3+^:^4^I_13/2_ levels of these four crystals were recorded upon direct excitation to Er^3+^:^4^I_11/2_ at room temperature and presented in Fig. 4.

The decay curves of the Er^3+^:^4^I_11/2_ level still fitted single-exponential decay well, which confirmed that quench effect and energy transfer processes had less influence on this level. On the contrary, the decay curves of the Er^3+^:^4^I_13/2_ level showed multi-exponential behavior, which confirmed that the lower laser level was greatly affected by the quench effect and energy transfer processes. Multi-exponential decay curves could be fitted with equation 2^29^,^30^:


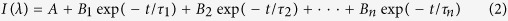


And then, the fluorescence lifetimes were calculated by equation 3^29^,^30^:


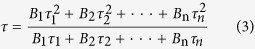


The fluorescence lifetimes of both laser levels (τ_1_, fluorescence lifetime of the upper laser level, τ_2_, fluorescence lifetime of the lower laser level) were listed in Table 2. Benefited from lower phonon energy, the τ_1_ of Er:SrF_2_ crystals were much longer than that of Er:CaF_2_ crystals. And longer lifetime of the upper laser level meant easier to achieve population inversion and better energy storage capacity. Even though τ_2_ were also extended in Er:SrF_2_ crystals, the values of Δτ/τ_1_ in Er:SrF_2_ crystals were still smaller than that of Er:CaF_2_ crystals.

With the MIR stimulated emission cross-sections and fluorescence lifetimes of Er^3+^:^4^I_11/2_ level the spectral quality factors (Q) were calculated and listed in Table 2. And the results were 7.45 × 10^−20^ ms*cm^2^, 3.89 × 10^−20^ ms*cm^2^, 4.83 × 10^−20^ ms*cm^2^, 2.62 × 10^−20^ ms*cm^2^, for 4at.% Er:SrF_2_, 4at.% Er:CaF_2_, 10at.% Er:SrF_2_, and 11at.% Er:CaF_2_ crystals, respectively. It was clear that in both SrF_2_ and CaF_2_, crystals with lower Er^3+^ doping level had larger spectral quality factors, which again indicated that 4at.% Er:SrF_2_ crystal might have better laser performance than 10at.% Er:SrF_2_. 4at.% Er:CaF_2_ had already been proven to have the best laser performance among a series of Er:CaF_2_ crystals in our previous work^3^.

Three crystals, namely 4at.% Er:SrF_2_, 10at.% Er:SrF_2_ and 4at.% Er:CaF_2_, were chosen in the following laser experiments. Thus, the laser properties of lightly doped and highly doped Er:SrF_2_ crystals could be compared. Further more, the laser properties of Er:SrF_2_ and Er:CaF_2_ crystals could also be compared.

### Laser experiments

**Figure 5:**
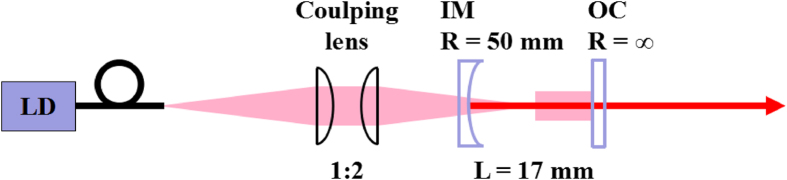
Schematic of the experimental setup for mid-infrared continuous-wave laser operation.

**Figure 6:**
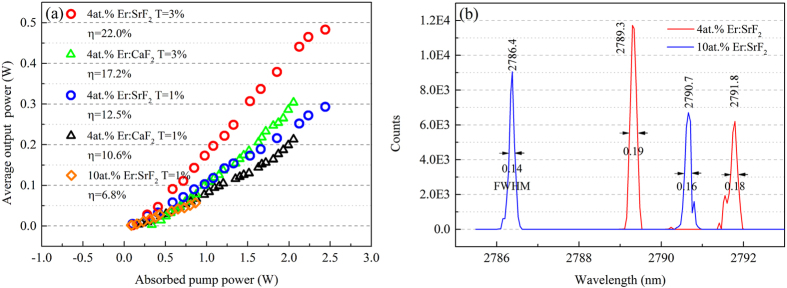
Laser operation of 4at.% Er:SrF_2_ 4at.% Er:CaF_2_ and 10at.% Er:SrF_2_, average output power versus absorbed pump power (**a**) and dual-wavelength of laser emission (**b**).

The CW laser experiments were carried out with a 17 mm long concave-plane laser resonator. The setup was shown in Fig. 5. Two output coupler with different output transmittance (T), T = 1% and T = 3% at 2.7–2.95 μm, were used to obtain the optimum laser output. The laser samples were mounted in a copper block and placed next to the output coupler. The copper block was kept at 10 °C by water cooling. Three uncoated laser samples were in dimensions of 3 × 3 × 10 mm^3^ for 4at.% Er:CaF_2_, 4at.% Er:SrF_2_ and 3 × 3 × 6 mm^3^ for 10at.% Er:SrF_2_. Finally, CW laser operations were demonstrated around 2.8 μm under 972 nm LD pumping, and the results were shown in Fig. 6. The average output power was measured by a power meter (30A-SH-V1, Israel).

As shown in Fig. 6a, CW laser operations around 2.8 μm were demonstrated in both lightly doped 4at.% Er:SrF_2_, 4at.% Er:CaF_2_ crystals and highly doped 10at.% Er:SrF_2_ crystal. Both 4at.% Er:SrF_2_ and 4at.% Er:CaF_2_ demonstrated better laser performance with the T = 3% output coupler than the T = 1% one.

Figure 6b showed that both 4at.% Er:SrF_2_ and 10at.% Er:SrF_2_ had dual-wavelength property, which was not found in Er:CaF_2_ crystals. The CW laser spectra were measured with an optical spectrum analyzer (MS3504i, SOL instruments, Belarus). Two laser wavelengths of 4at.% Er:SrF_2_ were 2789.3 nm and 2791.8 nm. Two laser wavelengths of 10at.% Er:SrF_2_ were 2786.4 nm and 2790.7 nm. And the FWHMs of laser spectra were less than 0.20 nm as marked in Fig. 6b.

4at.% Er:SrF_2_ crystal was proved to have the best laser performance among these three crystals, which was in agreement with the expectations based on spectral parameters. When T = 1%, the maximum output power of 0.293 W corresponding to a low threshold of 0.100 W was obtained with a slope efficiency of 12.5%, which was better than the slope efficient 11% of a 5at.% Er:SrF_2_ reported by T.T. Basiev^25^. When T = 3%, the slope efficiency was significantly improved to 22.0% with a better maximum output power of 0.483 W. And the threshold was maintained at 0.100 W, which was surely benefited from the low phonon energy and long fluorescence lifetime of 4at.% Er:SrF_2_ crystal.

4at.% Er:CaF_2_ crystal had worse laser performance than 4at.% Er:SrF_2_ crystal. When T = 1%, the maximum output power of 0.213 W corresponding to the threshold of 0.175 W was obtained with a slope efficiency of 10.6%. When T = 3%, the slope efficiency was improved to 17.2% with a better maximum output power of 0.304 W. However, the threshold also increased to 0.335 W, which was much higher than 4at.% Er:SrF_2_ crystal.

10at.% Er:SrF_2_ crystal only achieved CW laser operation with the T = 1% output coupler. Due to the degeneration of crystal quality and thermal conductivity, the maximum output power of 10at.% Er:SrF_2_ was only 0.057 W corresponding to a slope efficient of 6.8%.

These results confirmed the expectations based on spectral parameters that lightly doped 4at.% Er:SrF_2_ crystal was a promising candidate for low threshold, high slope efficiency mid-infrared lasers. Better results could be expected after optimizing the crystal growth and coating the samples.

## Conclusion

Compared with Er:CaF_2_ crystals, Er:SrF_2_ crystals had larger absorption cross-sections, larger MIR emission cross-sections, much longer fluorescence lifetimes and unique dual-wavelength laser properties. Lightly doped 4at.% Er:SrF_2_ crystal had better spectral parameters than both highly doped 10at.% Er:SrF_2_ crystal and Er:CaF_2_ crystals. As expected by spectral parameters, 4at.% Er:SrF_2_ crystal demonstrated the best laser performance with a low threshold of 0.100 W, a high slope efficiency of 22.0% and an maximum output power of 0.483 W. Hence, lightly doped 4at.% Er:SrF_2_ single crystal was a promising candidate for achieving for low threshold, high slope efficiency mid-infrared lasers.

## Additional Information

**How to cite this article**: Ma, W. *et al*. Highly efficient dual-wavelength mid-infrared CW Laser in diode end-pumped Er:SrF_2_ single crystals. *Sci. Rep.* **6**, 36635; doi: 10.1038/srep36635 (2016).

**Publisher’s note:** Springer Nature remains neutral with regard to jurisdictional claims in published maps and institutional affiliations.

## Figures and Tables

**Table 1:** The detailed values of α, σ_abs_ and FWHM of the 980 nm absorption bands.

Crystals	FWHM nm	α (cm^−1^)			σ_abs_ (×10^−20^ cm^2^)		
		971 nm	968 nm	972 nm	971 nm	968 nm	972 nm
4at.% Er:SrF_2_	20.01	1.58	—	1.53	0.198	—	0.192
4at.% Er:CaF_2_	24.43	—	1.76	1.48	—	0.181	0.153
10at.% Er:SrF_2_	19.42	3.80	—	3.67	0.174	—	0.168
11at.% Er:CaF_2_	23.07	—	4.48	3.42	—	0.174	0.132

**Table 2:** Fluorescence lifetimes (τ_1_, τ_2_), MIR emission cross-section (σ_em_) and spectral quality factors (Q) of 4at.% Er:SrF_2_, 10at.% Er:SrF_2_, 4at.% Er:CaF_2_ and 11at.% Er:CaF_2_ crystals at room temperature.

Crystals	τ_1_ (ms)	τ_2_ (ms)	Δτ/τ_1_	σ_em_ (10^−20^ cm^2^)	Q (10^−20^ ms*cm^2^)
4at.% Er:SrF_2_	9.56	15.06	0.58	0.78	7.45
4at.% Er:CaF_2_	5.98	9.94	0.66	0.65	3.89
10at.% Er:SrF_2_	7.43	10.03	0.34	0.65	4.83
11at.% Er:CaF_2_	4.86	8.25	0.69	0.53	2.62
